# The Obstetrician/Gynecologist

**Published:** 1994

**Authors:** John M. Thorp, Susanne Hiller-Sturmhöfel

**Affiliations:** John M. Thorp, Jr., M.D., is an assistant professor in the Division of Maternal-Fetal Medicine in the School of Medicine, University of North Carolina at Chapel Hill, North Carolina. Susanne Hiller-Sturmhöfel, Ph.D., is an associate editor for *Alcohol Health & Research World*

## Abstract

The obstetrician/gynecologist can play a pivotal role in the identification of alcohol-related problems in women. Direct intervention by the OB/GYN or referral to a specialized treatment program can initiate the recovery process for these women and prevent or improve many of their alcohol-related health problems.

The obstetrician/gynecologist (OB/GYN) is, in a sense, the primary health care provider for many women of all ages. These specialists see patients for annual routine examinations and pre- and postnatal visits and often serve as counselors for questions related to the women’s general health and reproduction. Some of these questions deal, directly or indirectly, with aspects and effects of the women’s alcohol consumption. Discussions about infertility, birth control, or pregnancy provide the OB/GYN with an opportunity to assess the patient’s alcohol consumption and to advise her about potential risks of alcohol use. In this way, necessary lifestyle changes can be initiated early, and problems in conceiving and delivering a healthy child, as well as other alcohol-related health problems, can be avoided.

For women with alcohol problems, the OB/GYN often makes the diagnosis of alcohol abuse or alcohol dependence and provides initial intervention. However, to counsel patients with drinking problems successfully, the OB/GYN must not only be able to identify alcohol abuse and alcohol-related problems but also must be familiar with different treatment and referral options.

This article describes alcohol-related problems encountered in an OB/GYN practice and the medical consequences associated with them. It also reviews screening tools available to the OB/GYN to assess alcohol consumption and addresses intervention options and considerations relevant to a successful treatment outcome.

## Kinds and Prevalence of Alcohol Problems

The Institute of Medicine (1990) defines alcohol problems as “problems that may arise in individuals around the use of beverage alcohol and that may require an appropriate treatment response for their optimum management” (p. 25). This very broad definition takes into account the extreme diversity of alcohol problems and their manifestations, whether social, physical, or psychological. It does not, however, include “indirect” consequences of alcohol abuse, such as physical or sexual abuse by an alcoholic spouse or partner. Although these problems may be present quite frequently among OB/GYN patients, they will not be addressed in the following discussion.

Problems encountered by the OB/GYN that are directly related to a woman’s alcohol use can range from the consequences of risky sexual behavior after alcohol consumption (e.g., unwanted pregnancy or sexually transmitted diseases) to severe physiological dysfunctions (e.g., irregular menstruation or infertility). An increased risk for breast cancer also has been associated with alcohol abuse (Longnecker et al. 1988; Rosenberg et al. 1993).

The prevalence of patients with alcohol-related problems in an OB/GYN practice is difficult to estimate. The diagnoses of alcohol abuse and alcohol dependence apply to 12 to 16 percent of OB/GYN patients (Halliday et al. 1986; Russell and Bigler 1979). And in a recent population-based survey, up to 25 percent of pregnant women reported alcohol consumption (Serdula et al. 1991).

## Medical Consequences of Alcohol Consumption

The predominant effects of alcohol consumption seen by the OB/GYN are related to problems in fertility, pregnancy, and the development of the fetus. A putative effect is an increased risk of breast cancer.

### Effects on the Endocrine and Reproductive Systems

Excessive^1^ alcohol consumption can reduce a woman’s fertility (Possati 1992; Gavaler 1991). Alcohol interferes with the female endocrine system and alters the amounts of reproductive hormones such as progesterone and estrogens (Gavaler 1991). Because of these changes, the menstrual cycle can become irregular or the menstrual period may be absent completely in chronic alcoholic women (Possati 1992; Becker et al. 1989). Excessive alcohol use also can lead to lack of ovulation, abnormalities of the ovaries, and even early onset of menopause (Possati 1992). The consequences of all these effects can range from infertility to an increased tendency for spontaneous abortions.

Women classified as “social drinkers” also exhibit some of these effects, although to a lesser extent (Possati 1992). The presence of endocrine and reproductive problems across such a wide range of women reinforces the importance of the OB/GYN being able to determine the drinking habits of all patients with fertility problems.

Alcohol also affects the endocrine system of postmenopausal women. Elevated levels of estrogens have been detected in both moderate and heavy drinkers (Gavaler 1991), although the consequences of this effect remain unclear.

### Effects on the Developing Fetus

Alcohol may interfere not only with a woman’s ability to conceive and carry a fetus to term but also with the development of the fetus, leading to fetal alcohol syndrome (FAS) and other alcohol-related birth defects (ARBD’s). The term “FAS” was first coined in the early 1970’s for a pattern of anomalies in children of alcohol-abusing mothers that includes poor fetal and postnatal growth, characteristically altered facial features, and developmental problems that persist into adulthood (Abel and Sokol 1991). ARBD, on the other hand, describes a variety of physical and neurobehavioral anomalies that can be attributed to the mother’s alcohol abuse but that do not meet all the criteria for FAS (Sokol and Clarren 1989).

Because of concerns about the effects of alcohol on the developing fetus, OB/GYN’s often are asked whether there is a “safe” amount of alcohol that can be consumed without harming the fetus. Given the variety of symptoms associated with FAS and other ARBD’s, it is impossible to determine a reliable threshold for safe alcohol consumption during pregnancy. Some neurobehavioral tests detect alcohol effects on fetal development even after extremely low levels of prenatal alcohol exposure (Jacobson and Jacobson 1994). Other studies have suggested that for effects such as delayed mental development, attention deficits, or lower IQ scores, the threshold level is more than seven drinks^2^ per week (Jacobson and Jacobson 1994). Because it is impossible to determine which babies may be at risk for damage from low levels of alcohol exposure, the Surgeon General has recommended that all women abstain from drinking throughout pregnancy (Public Health Service 1981).

Some alcohol-induced developmental deficiencies of the fetus can be avoided or ameliorated if the mother stops drinking during the pregnancy (Coles 1994). Therefore, it is important that the OB/GYN continues to monitor drinking women who may put the fetus at risk and advises them about the risks associated with their drinking behavior.

### Breast Cancer

Several studies indicate a higher prevalence of breast cancer in drinking women than in nondrinking women (for reviews, see Longnecker et al. 1988 and Rosenberg et al. 1993). How much the breast cancer risk is increased in drinking women varies from study to study. Generally, consumption of one drink daily is associated with a 1.4-fold higher prevalence compared with abstention. For women who drink two or more drinks per day, the risk of breast cancer is considered to be 1.8 to 2 times as high as for abstaining women.

How alcohol consumption increases the risk for breast cancer is not clear, and several possible mechanisms have been suggested (for a review, see Blot 1992). These include changes in hormone levels (e.g., estrogens), carcinogenic (i.e., cancer-causing) actions of alcohol degradation products such as acetaldehyde, induction of enzyme systems that can convert other substances into carcinogens, and alcohol effects on the liver that inhibit degradation of carcinogens.

## Identification of Drinking Problems

To identify alcohol problems accurately, the OB/GYN has to be aware that for each patient, both the drinking patterns and the resulting symptoms are different and can vary over time. Harmful drinking patterns most often encountered in the OB/GYN practice among nonpregnant women include binge drinking (five or more drinks on one drinking occasion) and heavy drinking (two or more drinks per day). As stated earlier, any alcohol consumption can be considered harmful for pregnant women. But even moderate drinking by nonpregnant women can lead to problems (e.g., risky sexual behavior). An accurate diagnosis may be further complicated because alcohol problems in women often are disguised as common psychiatric complaints such as anxiety or depression (Schmidt et al. 1990).

Several screening tools are available to the OB/GYN to help evaluate a woman’s drinking pattern and to determine whether a more thorough assessment of her alcohol consumption is warranted. These tools include questionnaire-based screening tests, laboratory tests for biological markers, and evaluations of physical symptoms.

### Questionnaire-Based Screening Tests

Screening questionnaires are the most practical and effective method of identifying patients with alcohol problems, and the OB/GYN can use them while taking a routine behavioral/medical history (see the article by Bradley, pp. 97–104). Questionnaire-based screening tests have been shown to detect problem drinkers with a high level of accuracy and efficiency.

One of the easiest and fastest screening tools used in primary care settings is the CAGE test (Ewing 1984; see Nilssen and Cone for the CAGE and also the TWEAK and T-ACE discussed below, pp. 136–139), which contains just four questions. The more extensive Michigan Alcoholism Screening Test (MAST; Selzer 1971) also is used frequently.

Two tests have been specifically designed for and successfully tested with pregnant women. The T-ACE (Sokol et al. 1989) was derived from the CAGE by including a tolerance question. The five-question TWEAK test (Russell et al. 1991) is composed of questions from the MAST, CAGE, and T-ACE tests that have been shown to be particularly sensitive to detecting alcohol abuse in women.

When administering these screening tests and interpreting their results, the OB/GYN must be aware that the patients are expected to admit to a potentially harmful behavior—drinking during pregnancy. They may be reluctant to report accurately, especially if the person administering the test appears to be judgmental and unsympathetic.

### Laboratory Tests and Physical Symptoms

Because of individual differences in the consequences of alcohol abuse, no laboratory test or physical symptom consistently identifies patients with alcohol problems. Consequently, the OB/GYN cannot rely solely on these methods to identify patients who abuse alcohol and require some kind of intervention.

For example, laboratory tests for biological markers of drinking consequences such as alcoholic liver disease, elevated enzyme levels, or anomalies of the blood cells usually identify only patients with severe alcohol problems (Halmesmaki et al. 1992). Similarly, physical symptoms such as hypertension, chronic headaches, insomnia, fatigue, or sexually transmitted diseases often do not occur in patients with less severe problems who could particularly benefit from early intervention (Holt et al. 1981).

## Intervention Options

Once OB/GYN’s have identified an alcohol problem in a patient, they must select the appropriate treatment. Options range from brief interventions offered during an office visit to referral to an alcoholism treatment specialist or a treatment program.

### Brief Interventions

The easiest intervention may be the initial screening test, particularly in patients with mild alcohol problems. Evidence suggests that participation in tests such as the CAGE or T-ACE may encourage lower alcohol consumption in some patients by increasing their awareness of their alcohol drinking patterns (Anderson 1993).

Office-based brief interventions such as provision of advice or self-help manuals or short counseling sessions are another option for the OB/GYN. Several studies have shown that these interventions may reduce the alcohol consumption of even moderate and excessive drinkers (see the article by Buchsbaum, pp. 140–145). Because these studies were done mostly in men, however, the effectiveness of brief interventions in the treatment of women has not been well established.

Brief interventions may not be sufficient for successfully treating women with severe social, medical, or psychological consequences of alcohol abuse. Given their need for extensive formal assessment and diagnosis and the wide spectrum of treatment programs available, referral to an alcoholism treatment specialist may be the most effective recommendation the OB/GYN can make.

### Referral to Treatment Specialists or Programs

When a referral is made, it is essential that both the referring physician and the alcoholism treatment specialist are supportive and encouraging toward the woman. Many people, including health professionals, disapprove of alcohol abuse in women more than they do in men (Zabolai-Csekme 1981; Vannicelli 1984; Gomberg 1993). Some health care professionals also consider women to be more difficult to treat and to have a poorer treatment outcome than men, although that belief has not been supported by scientific studies (Smith 1992; Gomberg 1993).

Because the therapist’s attitude toward women with alcohol problems is an important factor for the success of a treatment program (Gomberg 1993), careful selection and monitoring of the treatment specialist by the OB/GYN is important. Insensitive comments by a therapist such as “I never drank while I was pregnant” are hurtful to a patient who just accepted referral and can thwart the referring OB/GYN’s effort.

The OB/GYN also may refer alcohol-abusing patients directly to a treatment program. A plethora of treatment modalities is available, including residential programs such as therapeutic communities or drug treatment centers, outpatient programs of various intensities, and community-based programs such as Alcoholics Anonymous (AA) (Kumpfer 1991).

When selecting a program, the OB/GYN should consider special needs of women in treatment that will influence outcome. For instance, many women with drinking problems have a heavy-drinking partner (Zabolai-Csekme 1981; Smith 1992), and treatment programs should address this issue, for example through family therapy.

An important requirement for many women in therapy is the availability of child care (Wilsnack 1991). Treatment programs providing child care facilities, and perhaps even treatment options for the children of alcoholic mothers, could alleviate the patient’s guilt about leaving her children during treatment and her fear that because of her addiction, the children would be taken away from her (Wilsnack 1991). The referring OB/GYN can help by inquiring about treatment facilities’ child care options or by trying to arrange for the patient’s family members to take care of the children.

The OB/GYN also should evaluate AA or similar programs before referring patients. Although the universal availability and minimal costs make these programs a valuable treatment option, their efficacy for pregnant women has not been well documented. In addition, each group has its own characteristics, and the OB/GYN should try to find out whether local groups address issues particularly relevant to female patients (e.g., parenting, needs of battered women, or health questions related to pregnancy). Many AA chapters now have groups oriented toward women and welcome pregnant women and women with small children.

## Conclusions

OB/GYN’s are in a special position to identify women with alcohol problems and provide initial intervention. They see their patients regularly for routine examinations, often year after year. Discussions about reproductive issues provide opportunities to inform patients about the risks of alcohol use.

By incorporating simple screening tests into the routine history taking, OB/GYN’s can identify women with potential drinking problems early. Brief interventions or referral to professionals trained in assessing and treating women with alcohol disorders may be the first steps toward recovery for these patients.

## Footnotes

1 Because no studies have documented the exact level at which alcohol begins to cause deleterious effects on the endocrine system, the term “excessive” may correspond to a different consumption level for each individual.

2 One standard drink is defined as 12 fluid ounces of beer, 5 fluid ounces of wine, or 1.5 fluid ounces of distilled spirits.
